# Machine Learning Approaches for Measuring Neighborhood Environments in Epidemiologic Studies

**DOI:** 10.1007/s40471-022-00296-7

**Published:** 2022-06-30

**Authors:** Andrew G. Rundle, Michael D. M. Bader, Stephen J. Mooney

**Affiliations:** 1 Department of Epidemiology, Mailman School of Public Health, Columbia University, New York City, NY USA; 2 Department of Sociology, Johns Hopkins University, Baltimore, MD USA; 3 Department of Epidemiology, School of Public Health, University of Washington, Seattle, WA USA

**Keywords:** Machine learning, Neighborhood environments, Google Street View, Spatial interpolation, Neighborhood-wide association studies, Urban health informatics

## Abstract

**Purpose of review:**

Innovations in information technology, initiatives by local governments to share administrative data, and growing inventories of data available from commercial data aggregators have immensely expanded the information available to describe neighborhood environments, supporting an approach to research we call Urban Health Informatics. This review evaluates the application of machine learning to this new wealth of data for studies of the effects of neighborhood environments on health.

**Recent findings:**

Prominent machine learning applications in this field include automated image analysis of archived imagery such as Google Street View images, variable selection methods to identify neighborhood environment factors that predict health outcomes from large pools of exposure variables, and spatial interpolation methods to estimate neighborhood conditions across large geographic areas.

**Summary:**

In each domain, we highlight successes and cautions in the application of machine learning, particularly highlighting legal issues in applying machine learning approaches to Google’s geo-spatial data.

## Introduction

Neighborhood health research seeks to explain how neighborhood characteristics such as built features, social and economic conditions, and chemical and particulate pollutant concentrations affect residents’ health. In addition to public health and medicine, urban sociologists, planners, and architects contribute to the field. The methods used for study design and data analysis draw from sociology and environmental epidemiology. Findings from this body of research have influenced policymakers, architects, planners, and commercial entities, including supporting city policies that encourage health-promoting businesses like grocery stores and that establish urban design, architecture, and planning guidelines [1–3].

Defining and measuring neighborhood features presents challenges for neighborhood health effects research. Physical and social characteristics of neighborhoods vary widely and at multiple geographic scales in ways that make them difficult to characterize [4]. But innovations in information technology, the greater willingness of local governments to share administrative data, and a growing awareness of the types of data that can be purchased from commercial data aggregators have meant that the information available to characterize neighborhoods has expanded immensely over the past 20 years. These data have been linked to health data from surveys, health surveillance systems, schools, medical records, and epidemiologic studies [5–9]. We refer to urban health informatics as the use of information technology to tap into, organize, cross-link, and analyze the massive data stream produced by, and about, urban centers, to understand the health of residents [10]. Given the expanding data available for research, the field of neighborhood health research has started to use increasingly complex techniques to process these data more efficiently and accurately to characterize and identify exposures that affect health and health behaviors. Many of these techniques are drawn from machine learning, which we define here as the use of algorithms to uncover patterns in data that are then used without human intervention to make predictions about other data.

This review describes and evaluates three domains of urban health informatics in which innovative machine learning approaches have recently been applied (see Table 1). The first is automated image analysis of archived imagery such as Google Street View images. The second involves variable selection methods to identify neighborhood environment factors that predict health outcomes from large pools of exposure data with candidate variables. The third application uses spatial interpolation methods to estimate neighborhood conditions across large areas, using data collected at a limited number of sites.

**Table 1:** Summary of machine learning applications

Application	Uses	Issues
Automated image analysis	Accelerate virtual systematic social observation (VSSO) methods. Increase the density of sampled locations and expand the geographic coverage of VSSO studies	Legal issues with Google’s terms of use for Google Maps and Street View
Variable selection	Application of GWAS and EWAS-style studies to large pools of neighborhood-level variables	Choosing from the array of possible selection algorithms: results from different algorithms may disagree
Spatial interpolation	Characterize neighborhood conditions over large areas using data from a sample of locations	Researchers using “off-the-shelf” existing data versus researchers setting their own sampling plan for collecting data: need to account for uncertainty in the estimation of the neighborhood-level data

### Automated Image Analysis of Archived Imagery

The basis for much of the research on neighborhood health effects is derived from a method known as systematic social observation (SSO), also called neighborhood auditing [11, 12]. The method involves developing standard protocols to evaluate physical and social conditions (e.g., abandoned buildings, graffiti, and trash on the streets) on a systematic sample of locations (often street blocks or intersections) [11, 12]. The method was originally implemented by trained auditors visiting locations in person. In some cases, this involved auditing study participants’ neighborhoods when team members visited their homes to conduct face-to-face interviews or collect environmental samples, and in others, it involved auditing locations selected by the researchers to ensure geographic coverage of an area of research interest [11, 13].

SSO audit instruments have been developed and validated for measuring pedestrian safety features, pedestrian infrastructure, neighborhood physical disorder, advertising for alcohol and tobacco, and food environments. However, the in-person SSO approach has several limitations, including the time and expense for researchers to travel to the sampled locations, ensuring the physical safety of auditors, and community acceptance of researchers observing neighborhoods [14].

To address these problems, researchers developed virtual systematic social observations (VSSOs) [14, 15]. Instead of sending raters to physically inspect the block, VSSOs have trained auditors to use Google Street View’s archived imagery to collect observational data from streets or intersections [14, 16, 17]. Other implementations of VSSOs include collecting data from archives of images from public webcams [18]. Several existing in-person SSO protocols have been adapted for use in the virtual environment, and web-based tools have been developed to manage VSSO studies, notably the Computer-Aided Neighborhood Visual Assessment System (CANVAS) [19]. This approach has been shown to require much less time and expense than in-person audits while equivalently measuring the physical environment and offers the possibility of rating much larger geographic expanses, including national samples [16, 17, 19]. The strengths and weaknesses of VSSO have been previously discussed extensively [14–16].

**Fig. 1:**
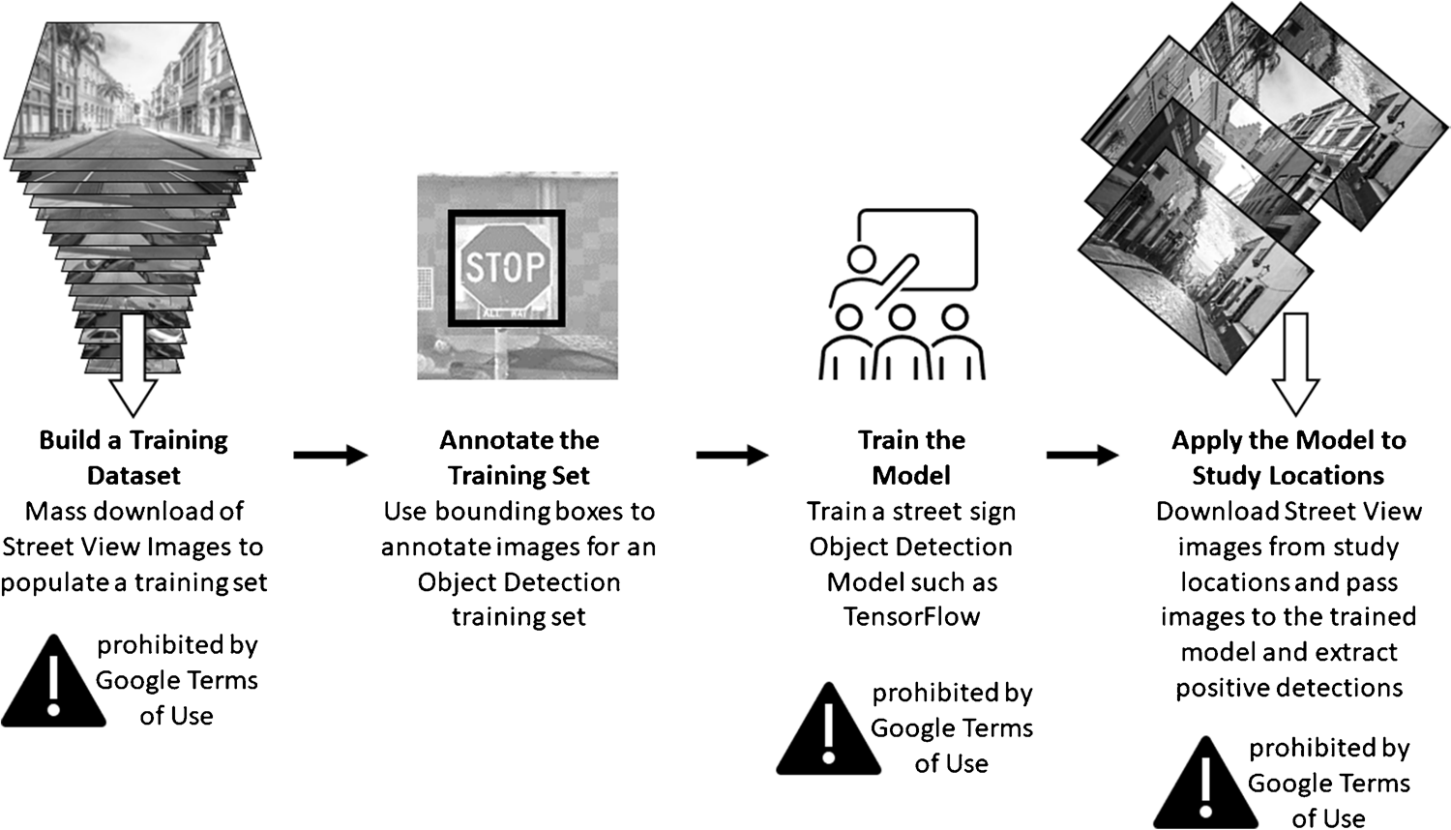
A common workflow for machine learning applied to Google Street View Images and its relationship to activities prohibited by Google’s terms of use [26]

Several groups have sought to expand the VSSO approach by replacing trained human observers with machine learning tools that automatically identify features in downloaded Google Street View images (see Fig. 1). This approach typically requires mass downloading of Street View images in order to efficiently process the images on high-performance computational clusters. There have been notable successes in training machines to quantify trees and green space and to identify traffic control signs, crosswalks, single-lane roads, and utility wires in Street View panoramas [20–26]. Recent work has also used health data from surveys to train a generative adversarial networks (GAN) machine learning model to identify health-related features in Street View images [27]. They found that respondents’ self-reported physical function using the PF-10 was associated with urban greenery, including tree height and building height identified in the images by the GAN model [27]. Another approach has been to have raters provide an evaluation of a single dimension, for example, safety, and then use the raters’ ratings to train machine algorithms to evaluate safety on other blocks [28]. Machine-learning-based approaches to identifying and quantifying key features of the built environment from Street View imagery have the potential to fully automate the conduct of VSSO and radically speed up the creation of data on neighborhood conditions.

We caution that machine learning approaches may ultimately incur legal challenges. As of January 2022—and since at least February 2018—Google’s overall terms of use and those of their Google Maps product (including “Geo Guidelines” included in the Google Maps terms) prohibit downloading and storing imagery, recreating panoramic views from downloaded image tiles, and the use of “…applications to analyze and extract information from the Street View imagery” (see Fig. 1). [29, 30] The creation of measures of trees from Google Maps products is specifically given as an example of prohibited uses [29]. The terms of use also prohibit the use of Google Maps-derived data in point-in-polygon analyses, a geographic information systems technique commonly used in neighborhood health effects research. The Geo Guidelines explicitly state that nonprofit and academic uses are not exempt from the terms: “these restrictions apply to all academic, nonprofit, and commercial projects,” and further that they will not grant exceptions: “If your use is not allowed, we are not able to grant exceptions, so please do not submit a request.”[30] These prohibitions are not based on copyright, for which researchers might invoke the concept of fair use, but based on the contract a user agrees to follow (an “end user’s licensing agreement”) by accessing Google Maps. [31] The enforceability of such contracts is an area of active litigation and, as such, is unclear; researchers who conduct this type of research and the journals that publish the resulting papers incur some legal risk so long as the law remains unsettled [8, 32].

### Variable Selection Approaches to Analyzing “Big Data”

A second area of innovation regarding machine learning and neighborhood health research involves variable selection in prediction models using “hypothesis-free” analyses. Vast amounts of data can be processed and linked together using geographic information systems (GIS), including census data, business listing data (e.g., the National Establishment Times Series), social media data, online search data, and administrative data from local, state, and federal governments (e.g., tax, licensing, inspection, maintenance, and enforcement data) to measure neighborhood conditions [33–35]. When all of these data sources are compiled together, the GIS becomes a high-throughput data-generating platform that can produce thousands of variables describing the environment of a neighborhood.

**Fig. 2:**
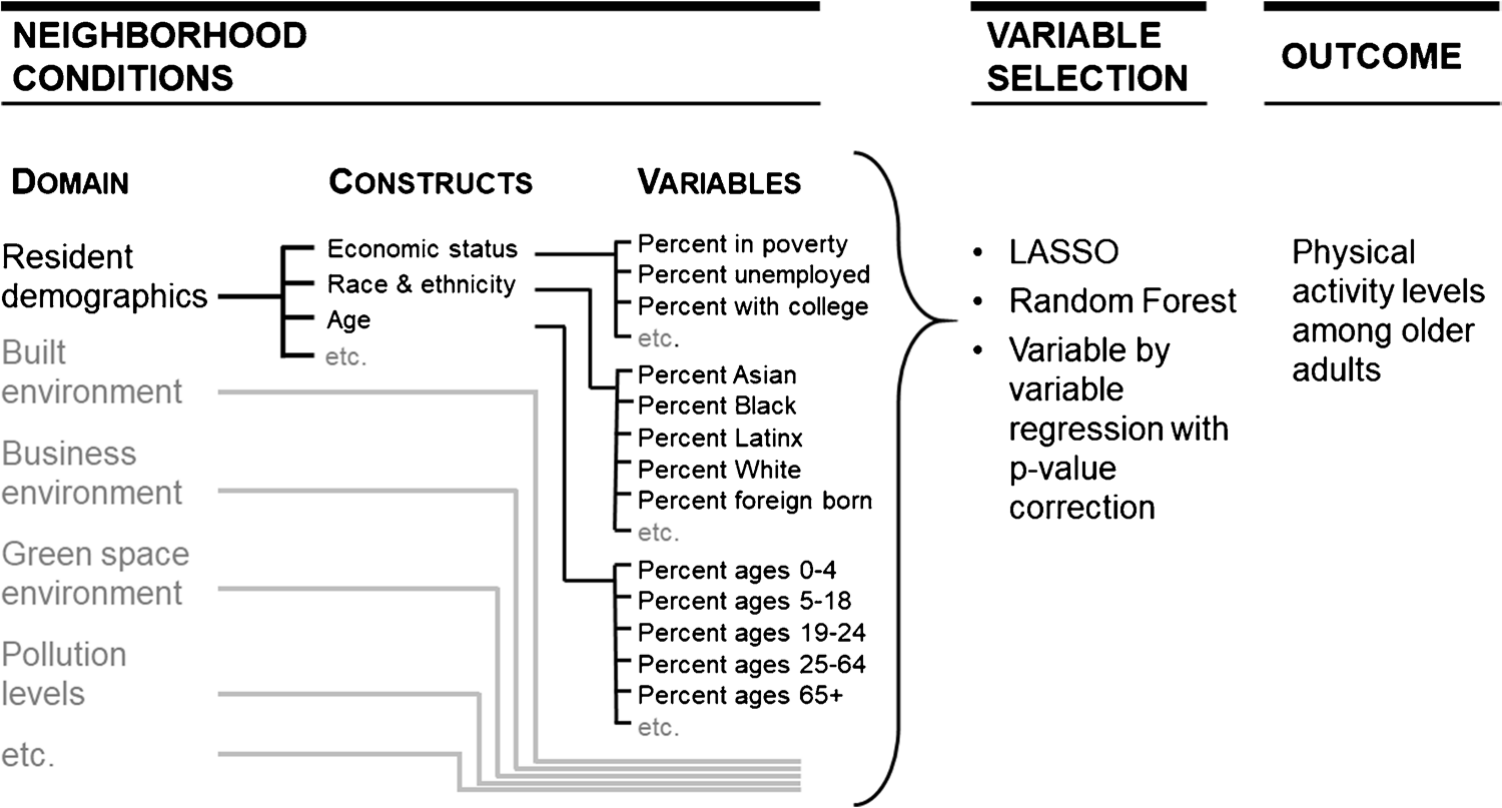
Schematic diagram of a neighborhood environment-wide association study as applied to selecting variables that predict physical activity levels in older adults [41]

While the quantity and richness of these data are very attractive to researchers, the variables are often highly correlated, causing multicollinearity issues in multivariable data analyses that limit the ability to isolate the effects of individual variables using hypothesis-based approaches to data analysis (see Fig. 2). As in other fields that have faced these issues, like with the -omic arrays used to study genomics and proteomics, and studies of exposures to complex chemical mixtures, researchers studying neighborhood health effects have turned to machine learning and variable selection approaches to identify neighborhood environment variables associated with health outcomes [36–38]. Using genome-wide association studies (GWAS) [39] and environment-wide association studies (EWAS) [40] as a template, neighborhood health researchers have begun implementing neighborhood environment-wide association studies (NE-WAS) [41–44]. These studies use computer algorithms to identify neighborhood environment variables most strongly associated with health outcomes of interest [41]. Examples of this are efforts to identify neighborhood-level variables that predict prostate cancer aggressiveness, physical activity levels among older adults, COVID-19 mortality, neighborhood-based walking, and violent crime [41, 42, 44–46].

How best to conduct NE-WAS analyses remains unclear. For any of the ‘-WAS approaches, including NE-WAS, there are numerous algorithms for simultaneously analyzing large quantities of predictor variables, and researchers debate which of these algorithms identifies relationships of greatest scientific interest [36–38, 47–50]. Simple approaches include calculating an odds ratio and *p*-value for each predictor variable independently and applying a multiple comparisons correction to the threshold for declaring statistical significance [41, 42]. Other approaches, which are vastly more computationally complex, include reducing the number of dimensions (e.g., through principal components, latent classes, *k*-means clustering, etc.) and variable selection steps (e.g., LASSO or stepwise regression) [38, 47, 48, 50]. These approaches often sub-set the full dataset into training and validation subsets and then use cross-validation to select tuning parameters from the training subset, validate the parameters on the validation subset, and then apply the parameters to the entire dataset [38, 47, 48, 50].

These complex analytic techniques, developed by computer data scientists, make principled use of patterns in the data to build robust classification and prediction models. However, their successful use in neighborhood research has been more limited. One reason for this is that algorithms designed to select the variables that together best predict an outcome do not, in general, select a subset of distinct variables that are most causally relevant [51]. Moreover, because neighborhood predictors are frequently strongly correlated with each other (e.g., % of households living in poverty is highly correlated with median household income), naive application of variable selection algorithms (e.g., with no pre-selection of variables to ensure measures cover only a subset of domains) results in highly unstable selections—minor tweaks to the dataset result in a very different selection of variables [52].

To address a similar issue of highly correlated predictor variables in GWAS studies, an initial process of “pruning” is often used to remove from a dataset the data for one or the other of two genetic loci with SNPs that show high linkage disequilibrium (high correlation) [53–55]. Typically, in pruning GWAS data sets, the data for the loci with the highest minor allele frequency (MAF) is kept in the dataset, and the loci with the lowest MAF are removed from the dataset [53–55]. Because neighborhood-level variables are commonly continuously distributed, there is not an exact analogy to MAF to guide pruning decisions in NE-WAS studies. However, considerations related to the effects of measurement error on bias in neighborhood health effects studies can be used to guide decisions regarding which variables to prune. Measurement error in underlying data (e.g., personal income data collected in the American Community Survey) that are aggregated up to create neighborhood-level measures (e.g., % of the population living in poverty or median household income or per capita income) causes systematic bias away from the null in neighborhood health effects studies when the neighborhood level variable is expressed as a proportion (e.g., % of the population living in poverty) but not for variables expressed a continuous scales (e.g., median household income) [56]. Thus, for variable pruning in NE-WAS studies, within sets of highly correlated variables, we recommend removing variables expressed as proportions.

### Spatial Interpolation to Estimate Neighborhood Conditions

Spatial interpolation is another area where machine learning approaches have been applied to neighborhood health research, including in studies of air pollution, of neighborhood physical disorders, and sidewalk conditions [4, 57–59]. Spatial interpolation models such as kriging and land use regression estimate neighborhood conditions at all locations across a geographic area using measured data from only a sample of locations in the region [4, 57, 60–63]. The estimates are based on the measured values at the sampled locations, distances to sampled points, and the spatial correlation between measured values at sampled points. The estimation models often also include external data measured at all locations in the target geographic area (e.g., home prices or distance to roads). Spatial interpolation can use machine learning techniques, including automated variable selection techniques that can select predictors and functional forms included in a final model from a class of candidate predictors and leave-one-out cross-validation that “tunes” the interpolation model [64].

Spatial interpolation can be implemented using several approaches. One such approach is land use regression, which is commonly used in air pollution studies. This method uses data on relevant land use features (e.g., industrial zoning, road density, vehicle traffic, and pollution point sources) to create a regression model predicting pollution levels measured at air monitoring stations [60, 61, 65]. This regression model is then used to estimate air pollution at all other locations in the target area [60].

Another spatial interpolation approach is ordinary kriging, which uses the spatial correlation between the variable values at each sampled location (e.g., particulate matter measured at air monitors) to estimate values at all non-sampled locations in the geographic area of interest [4, 57]. Universal kriging is an extension of ordinary kriging that uses additional external data that can be measured at all locations in the geographic area to supplement the information represented by the spatial correlations [66]. Universal kriging can be viewed as jointly modeling ordinary kriging and land-use regression [60]. In a study of particulate matter air pollution, for example, relevant external data might be vehicle traffic volume. In our work using universal kriging to estimate neighborhood physical disorder in four US cities, we found that using a measure of housing vacancy in universal kriging improved estimation over ordinary kriging for Philadelphia and Detroit. [66] More complex approaches apply automated techniques to a suite of candidate variables to improve these models, either by algorithmically selecting specific environment variables to include in a model or by applying dimensionality reduction techniques to identify underlying factors that maximize the predictive value of external data [64, 67]. These complex approaches are more commonly used to characterize exposures to environmental pollutants than neighborhood built or social environment factors, but they present a promising direction for new methods to describe neighborhood conditions.

There are several considerations to be acknowledged when using spatial interpolation techniques. When data are available from a series of locations “off the shelf” (often the case for air monitoring data but can also be true for other administrative data), the spatial distribution of these locations is often not optimized for making interpolation estimates at non-sampled locations [57]. In these off-the-shelf scenarios, the estimates can have greater uncertainty than would be seen if the sampling were designed to optimize spatial interpolation. In air pollution studies, the air monitoring stations set up for administrative purposes are often located in such a way that there is higher uncertainty for the interpolated estimates near the borders of a city or region [57]. Spatial interpolation works best when the sample of locations where data will be collected is chosen to optimize spatial interpolation algorithms [60, 65]. There are considerations when researchers choose sample locations to collect data from; should the sample be based on population distribution of land area [4]? When chosen sampled locations to reflect population distribution in an area, the resulting sample will on average represent the population but not necessarily geography and land uses [4]. For example, non-inhabited but relevant areas like industrial plants or parks would be excluded from a sample of locations selected based on population distribution.

When interpolated neighborhood exposure values are used in a regression analysis predicting some health outcomes, the uncertainty in the neighborhood measurements should be included in analyses of health outcomes. Not doing so will lead to underestimating the uncertainty of parameter estimates measuring the association between the neighborhood exposure and the health outcome. A solution to this issue uses a framework very similar to multiple imputations for missing data [4, 58]. Instead of a single value being estimated for each neighborhood location of interest, multiple values for each location are estimated from the interpolation model to represent the uncertainty in the estimation process [4, 58]. These multiple exposure data sets are then each analyzed to predict the health outcome and the estimated effect size and its corresponding standard error from each data set are combined to create a pooled estimated effect size and standard error. As in multiple imputations, the pooled standard error from analyzes of multiple estimated data sets better expresses the uncertainty in the observed association between exposure and outcome [68].

## Conclusion

Revolutions in information technology, the greater willingness of local governments to share their administrative data, and a growing awareness of the types of data that can be purchased from commercial data aggregators mean that the information available to characterize neighborhoods has expanded immensely over the past 20 years. As data availability has expanded, researchers studying neighborhood health effects have started to utilize machine learning approaches to measure and identify neighborhood features that influence health. Spatial interpolation methods, particularly for estimating air pollution, have the most established track record for the use of machine learning in characterizing neighborhood environments. While GWAS have been employed for over twenty years in genetic epidemiology, similar variable selection approaches have just begun to be implemented with neighborhood-level data. In other fields that grapple with large quantities of intercorrelated predictor variables, such as -omics and the study of chemical mixtures, there is debate over which variable selection algorithms are most appropriate. Lessons learned from these other fields are likely to be applicable to neighborhood-level data.

Of the machine learning approaches that have been used, it is the application of automated image analysis that has perhaps most captured the imagination of researchers. Unfortunately, the terms of use for Google Street View, the data source most commonly used for VSSOs, expressly prohibit the use of machine learning to identify features in Street View images. We hope that ongoing litigation will clarify the enforceability of terms of use and that companies that create these valuable spatial data sets will make them available for health research. Yelp, for example, has a special program for academic researchers who wish to use their data[69]. Until then, journals and Institutional Review Boards should pay attention to the use of data acquired without appropriate licenses or in ways contrary to the terms of use.
